# Comparison of Heterosubtypic Protection in Ferrets and Pigs Induced by a Single-Cycle Influenza Vaccine

**DOI:** 10.4049/jimmunol.1800142

**Published:** 2018-04-27

**Authors:** Barbara Holzer, Sophie B. Morgan, Yumi Matsuoka, Matthew Edmans, Francisco J. Salguero, Helen Everett, Sharon M. Brookes, Emily Porter, Ronan MacLoughlin, Bryan Charleston, Kanta Subbarao, Alain Townsend, Elma Tchilian

**Affiliations:** * The Pirbright Institute, Pirbright GU24 0NF, United Kingdom;; † Laboratory of Infectious Diseases, National Institute of Allergy and Infectious Diseases, National Institutes of Health, Bethesda, MD 20814;; ‡ School of Veterinary Medicine, University of Surrey, Guildford GU2 7AL, United Kingdom;; § Animal and Plant Health Agency, Weybridge, New Haw, Addlestone, Surrey KT15 3NB, United Kingdom;; ¶ School of Veterinary Sciences, University of Bristol, Langford, Bristol BS40 5DU, United Kingdom;; ‖ Aerogen Ltd., Dangan, Galway H91 HE94, Ireland; and; # Weatherall Institute of Molecular Medicine, University of Oxford, Headington, Oxford OX3 9DS, United Kingdom

## Abstract

Influenza is a major health threat, and a broadly protective influenza vaccine would be a significant advance. Signal Minus FLU (S-FLU) is a candidate broadly protective influenza vaccine that is limited to a single cycle of replication, which induces a strong cross-reactive T cell response but a minimal Ab response to hemagglutinin after intranasal or aerosol administration. We tested whether an H3N2 S-FLU can protect pigs and ferrets from heterosubtypic H1N1 influenza challenge. Aerosol administration of S-FLU to pigs induced lung tissue-resident memory T cells and reduced lung pathology but not the viral load. In contrast, in ferrets, S-FLU reduced viral replication and aerosol transmission. Our data show that S-FLU has different protective efficacy in pigs and ferrets, and that in the absence of Ab, lung T cell immunity can reduce disease severity without reducing challenge viral replication.

## Introduction

Influenza virus infection is a global health threat to livestock and humans, causing substantial mortality. The major obstacle in combating influenza is the rapid evolution of the virus, rendering the host Ab response ineffective. Seasonal influenza virus vaccines are therefore strain specific, do not protect well against drifted viruses from the same hemagglutinin (HA) subtype, and offer no protection against infection with heterologous influenza viruses from different HA subtypes. Furthermore, pandemic influenza can arise at any time, originating from either group 1 or 2 avian influenza A viruses (IAV) and can cause devastating mortality. Therefore, a broadly protective influenza A vaccine (BPIV), which could protect against both group 1 and 2 viruses, would be a great advance in preventing seasonal infection and reducing mortality from pandemic influenza (1).

Signal Minus FLU (S-FLU) is a replication-incompetent influenza virus, candidate BPIV, and is limited to a single cycle of replication (2) through inactivation of the HA signal sequence (3). Functional HA protein, which is required to form infectious virus particles, is provided in trans from a transfected cell line by pseudotyping, and the S-FLU vaccine virus can therefore infect the host but cannot replicate. All of the conserved viral core proteins are expressed in the cytosol of S-FLU–infected cells for optimal Ag presentation to T lymphocytes (4). S-FLU induces a strong cross-reactive T cell response in the lung to the conserved core proteins, a specific Ab response to the expressed neuraminidase (NA), but a minimal Ab response to the HA coating the particle when administered to the respiratory tract. Immunization of mice with H1N1 or H5N1 S-FLU results in a high degree of protection against the homologous and heterologous H1N1, H6N1 (group 1), H3N2, and H7N9 (group 2) viruses, with moderate protection against distinct (heterologous) H5N1 (3, 5). Similarly, in ferrets, immunization with H1N1 or H5N1 S-FLU significantly reduced replication of H1N1, H6N1, H5N1 (group 1), and H7N9 (group 2) viruses in the lung. In pigs, immunization with H1N1 or H5N1 S-FLU reduced the viral load in nasal swabs and lungs following challenge with a swine H1N1pdm09 isolate (6).

Because S-FLU can neither replicate nor donate its HA sequence to other influenza strains if administered to infected individuals, it should be safe. For this reason and because immunization via the lower respiratory tract has been shown to be a highly effective means of immunizing against influenza, in all experiments with S-FLU, the vaccine was administered either intranasally to mice and ferrets or intranasally, intratracheally, or by aerosol in pigs. Our results show also that targeting the lower respiratory tract by aerosol in pigs is more effective than intratracheal or intranasal immunization in preventing severe disease (6). The reason for this may be that local immunization induces lung tissue-resident memory T cells (T_RM_), which have been shown to be important in cross-protective immunity against influenza infection (7–10). Most work on T_RM_ has been performed in mice and the T_RM_ defined as inaccessible to intravenously administered anti–T cell Ab (11). T_RM_ identified in this way are an activated, dividing population capable of responding rapidly to Ag by further cell division in situ in mice. However, there are very few data on T_RM_ in large animals.

To further assess whether S-FLU vaccines could protect from a completely heterosubtypic challenge, in which the HA and NA of the vaccine and challenge viruses belonged to different genetic groups, we have tested the protective efficacy of an S-FLU coated in contemporary human H3N2 (group 2) glycoproteins against challenge with an H1N1pdm09 (group 1) virus in both pigs and ferrets.

## Materials and Methods

### Vaccines and influenza challenge virus

The design and production of pdmH1N1 S-FLU [eGFP*/N1(Eng195)].H1(Eng/195/2009) has been described previously (3, 5). We made a new H3N2 S-FLU [eGFP*/N2(×217)].H3/SW/9725293/2013 (encoding N2 from A/Victoria/361/2011 from the vaccine strain ×217 and coated in H3 from A/Switzerland/9725293/2013) at 1.52 × 10^8^/ml 50% tissue culture infectious doses (TCID_50_) (95% CI 1.13 to 2.05 × 10^8^/ml). The internal protein gene segments were from influenza A/Puerto Rico/8/34 (H1N1).

In the ferret studies, a live attenuated influenza A/Switzerland/9715293/2013 cold-adapted (ca) vaccine on the influenza A/Ann Arbor/6/60 ca backbone was included as a comparator, and a group of mock-immunized control animals received Leibovitz 15 (L-15) media. Influenza A/California/07/2009 (H1N1pdm09) and A/Switzerland/9715293/2013 (H3N2) viruses were used for challenge infection.

The pig isolate of A/swine/England/1353/09pdmH1N1 (1353/09pdmH1N1) was used for challenge infection in pigs. The homologous vaccine consisted of the identical β-propiolactone inactivated 1353/09pdmH1N1 with TS6 adjuvant. TS6 adjuvant was kindly provided by Dr. Catherine Charreyre (Merial/Boehringer Ingelheim). It contains an oily phase (comprising sorbitan monooleate, sorbitan trioleate, paraffin oil, and sodium mercurothiolate) and an aqueous phase of monopotassium and disodium phosphate.

### Aerosol characterization

We first established that passage through the Aerogen Solo vibrating mesh nebulizer (Aerogen, Dangan, Galway, Ireland) did not significantly reduce the titer of S-FLU. The cell supernatant containing S-FLU in viral growth medium (DMEM/0.1% BSA/10 mm HEPES pH 7) was passed through a 0.22 μm filter then aerosolized using the nebulizer, captured, and condensed. The effect of nebulization on the infectious titer of S-FLU was measured on three different batches of S-FLU coated in three different HAs by comparison of quadruplicate measurements of the means of the number of doubling dilutions (i.e., Log2 of the dilution factor) giving 50% infection of MDCK-SIAT cells (calculated by linear interpolation) pre- and postnebulization by an unpaired *t* test (Prism v7.0). H5 (A/Vietnam/1203/2004) −0.2938 (95% CI: −0.5059 to −0.08161, *p* = 0.0147) = 18.4% reduction; H7 (A/Netherlands/219/2003) −0.2067 (95% CI: −0.5808 to 0.1674, *p* = 0.225) = 13.3% reduction; H3 (A/Victoria/361/2011) −0.04246 (95% CI: −0.2699 to 0.185, *p* = 0.664) = 2.9% reduction. Although the 18.4% reduction in the titer of the H5 S-FLU after nebulization was statistically significant, the minimal effects on the H7 and H3 batches did not reach statistical significance. We regard these small effects of the vibrating mesh nebulizer on infectious titer of S-FLU as not biologically significant.

We then assessed the aerosol droplet size distribution using a cascade impactor (Next Generation Impactor; Copley Scientific) at 15 l/min vacuum flow rate. A known quantity of virus (2.13–3.39 × 10^9^ CID_50_ in 4 ml viral growth medium) was passed into the impactor and subsequently harvested from each of the impactor stages, which fractionate the aerosol droplets by size. In three replicates, the mean aerodynamic size of the aerosol droplets was 1.953 μm with a geometric SD of 1.795. The fine particle fraction, which is the fraction of the aerosol produced with a droplet size <5 μm, was 92.34%, indicating that the aerosol produced was highly respirable.

### Animals and immunizations

#### Pigs.

All experiments were approved by the ethical review processes at the Pirbright Institute and Animal and Plant Health Agency and conducted according to the U.K. Government Animal (Scientific Procedures) Act 1986. Both institutes conform to Animal Research: Reporting of In Vivo Experiments guidelines. Eighteen, 5–6-wk-old landrace cross female pigs were obtained from a commercial high-health status herd (average weight of 10 kg at the beginning of the experiment). Pigs were screened for absence of influenza A infection by matrix gene real-time RT-PCR and for Ab-free status by hemagglutination inhibition using four swine influenza virus Ags. Pigs were randomly divided into three groups of six and immunized as follows: 1) control unimmunized, 2) Homologous (1353/09pdmH1N1) vaccine containing 2048 hemagglutination units administered i.m. in 1 ml, and 3) H3N2 S-FLU administered by aerosol delivering ∼1.5 × 10^8^ TCID_50_ in 1 ml. The animals received an identical booster immunization 21 d later. S-FLU was administered using an Aerogen Solo nebulizer attached to a custom-made mask held over the animal’s nose and mouth (Supplemental Fig. 1) following sedation.

For logistical reasons, two IAV challenges were performed, with half of the animals challenged at 28 d postboost (dpb) and the remainder at 30 dpb. Animals were challenged intranasally with 1.5 × 10^6^ PFU per pig of 1353/09pdmH1N1. Two milliliters were administered to each nostril using a mucosal atomization device, MAD300 (Wolfe Tory Medical). As the analysis of samples from pigs challenged at days 28 and 30 did not show any significant differences, for simplicity in presentation, the results of the assays carried out on pigs challenged on both days have been amalgamated in all figures.

#### Ferrets.

Four- to six-month-old female ferrets that were seronegative by hemagglutination inhibition assay to circulating influenza A H1N1pdm09 and H3N2 viruses were purchased from Triple F Farms, Sayre, PA. The ferret experiments were conducted in animal BSL2 laboratories at the National Institutes of Health in compliance with the guidelines of the Institutional Animal Care and Use Committee. Ferrets were lightly anesthetized with isoflurane and immunized intranasally with two doses of 10^7^ TCID_50_ in 0.5 ml of A/Switzerland/9715293/2013 S-FLU, 10^7^ TCID_50_ in 0.5 ml of A/Switzerland/9715293/2013 ca, or 0.5 ml of L-15 21 d apart. The ferrets were challenged with 10^6^ TCID_50_ in 1 ml of influenza A/California/07/2009 (H1N1pdm09) or A/Switzerland/9715293/2013 (H3N2) virus.

### Pathological and histopathological examination of pig lungs

Animals were humanely killed 5 d postchallenge (dpc). At post mortem, the lungs were removed, and digital photographs were taken of the dorsal and ventral aspects. Macroscopic pathology was scored blind, as previously reported (12). Five lung tissue samples per animal from the right lung (two pieces from the apical, one from the medial, one from the diaphragmatic, and one from the accessory lobe) were collected into 10% neutral buffered formalin for routine histological processing at the University of Surrey. Formalin-fixed tissues were paraffin wax–embedded, and 4-μm sections were cut and routinely stained with H&E. Immunohistochemical staining of influenza virus nucleoproteins (NP) was performed in 4-μm tissue sections as previously described (13). Histopathological changes in the stained lung tissue sections were scored by a veterinary pathologist blinded to the treatment group. Lung histopathology was scored using five parameters (necrosis of the bronchiolar epithelium, airway inflammation, perivascular/bronchiolar cuffing, alveolar exudates, and septal inflammation) scored on a five-point scale of 0 to 4 and then summed to give a total slide score ranging from 0 to 20 and a total animal score from 0 to 100 (6). The Iowa system includes both histological lesions and immunohistochemical staining for NP (14).

### Tissue sample processing

#### Pigs.

Four nasal swabs (two per nostril) were taken daily after the challenge. Serum and heparin anticoagulated blood samples were collected at the start of the study (prior to immunization) and at days 7, 14, 21, 28, 35, 42, and 49 after the first immunization. Bronchoalveolar lavage (BAL) and tracheobronchial lymph nodes (TBLN) were processed as previously described (6). Medial and diaphragmatic lung cells were dissociated into a single-cell suspension with the gentleMACS Octo Dissociator (Miltenyi Biotec), using C tubes (Miltenyi Biotec) with 5 ml of serum-free RPMI 1640 containing collagenase and DNAse (Sigma-Aldrich). Following dissociation, the tubes were incubated at 37°C for 20 min, the resulting suspension was mashed through a tea strainer using complete RPMI 1640, and the single-cell suspension was filtered twice using a 100 μm cell strainer, washed, and RBCs were lysed. Cells were washed and cryopreserved.

#### Ferrets.

Four ferrets from each group were sacrificed at 2 and 4 dpc, and their lungs and nasal turbinates (NT) were harvested. Harvested tissues were homogenized in L-15 medium at 10% (w/v) for lung or 5% (w/v) for NT samples clarified by centrifugation at 2500 rpm for 10 min.

### Transmission studies in ferrets

We performed airborne transmission studies using a caging system previously described (15). Briefly, four adult ferrets obtained from Triple F Farms that were seronegative by hemagglutination inhibition to circulating H1N1pdm09 and H3N2 viruses were anesthetized by i.m. injection of a ketamine-xylazine mixture prior to intranasal immunization with two doses of H3N2 S-FLU or with L-15 medium alone (mock immunized). Twenty-one days after the second dose, ferrets were challenged with 10^6^ TCID_50_ of A/California/07/2009 virus. Challenged ferrets were placed into the section of the cage closest to the air inlet the day of challenge. One day later, a naive ferret was placed into the cage on the other side of the divider. Environmental conditions inside the laboratory were monitored daily and were consistently 19 ± 0.3°C and 60 ± 2.2% relative humidity. The transmission experiments were conducted in the same room to minimize any effects of caging and airflow differences on aerobiology. The naive ferret was always handled before the infected ferret. Animals were carefully handled during nasal wash collections and husbandry to ensure no direct contact occurred between the ferrets. Nasal washes were collected every other day for 14 d and analyzed for the presence and titer of infectious viruses and expressed as TCID_50_ per ml. On day 14 postinfection, blood was collected from each animal, and serology was performed by hemagglutination inhibition and microneutralization assays.

#### Virus titration.

Viral titers in nasal swabs and BAL from pigs were determined by plaque assay on MDCK cells as previously described (6). Clarified homogenates of ferret tissues were titrated on MDCK cell monolayers, and virus titers were calculated by the Reed and Muench method and expressed as log_10_ TCID_50_ per g.

#### Microneutralization assay.

Neutralizing Ab titers in serum were determined as previously described (3, 16)

#### IFN-γ ELISPOT assay.

Frequencies of IFN-γ–secreting pig PBMC and BAL cells were determined by ELISPOT assay using fresh or cryopreserved cells (6). Cells were stimulated with either 1 × 10^6^ PFU live MDCK-grown1353/09pdmH1N1, 1 × 10^5^ TCID_50_ H3N2 S-FLU, medium control, or 10 μg/ml Con A (Sigma-Aldrich). Results were expressed as number of IFN-γ–producing cells per 10^6^ cells after subtraction of the average number of IFN-γ^+^ cells in medium control wells.

#### Flow cytometry.

Cryopreserved mononuclear cells from blood, TBLN, BAL, spleen, and lung were thawed and stimulated for 12 h at 37°C with either 1 × 10^6^ PFU live MDCK-grown strain 1353/09pdmH1N1 or 1 × 10^6^ TCID_50_ H3N2 S-FLU or MDCK mock supernatant as control. GolgiPlug (BD Biosciences) was added for a further 4 h before intracellular cytokine staining. Cells were stained for surface markers with CD3ε-AF647 BB23-8E6-8C8, CD4 clone 74-12-4 PerCpCy5.5, CD8α-FITC 76-2-11 (BD Biosciences), and Near-Infrared Fixable LIVE/DEAD stain (Invitrogen). Cells were permeabilized using Cytofix/Cytoperm (BD Biosciences) before intracellular staining with IFN-γ PE P2G10 (BD Biosciences) and cross-reactive anti-human TNF-α-AF650 Mab11 (BioLegend). Samples were fixed in 1% paraformaldehyde before analysis using an LSRFortessa (BD Biosciences). Data were analyzed using FlowJo v10 (Tree Star).

#### Lung T_RM_.

Before sacrifice, three animals from each group were infused i.v. with 10 ml of 3.24 mg/ml purified CD3 Ab (clone PPT3) and sacrificed 10 min later. Lymphocytes were isolated and stained ex vivo with anti-mouse Ig-FITC (SouthernBiotech), which labels the circulating intravascular cells. The cells are washed, and normal mouse serum is then added to block any remaining binding capacity of the anti–Ig-FITC. The cells are then washed again and incubated with CD3 Ab labeled with PeCy5 (Abcam). This will bind unsaturated sites of the circulating cells, which are therefore double labeled, as well as all the sites on the T_RM_ that are not accessible to the CD3 Ab, given i.v. T_RM_ is therefore single labeled with PeCy5.

To allow intracytoplasmic staining of T_RM_, the i.v. CD3 was detected with goat anti-mouse IgG BV421 (BioLegend) and blocked using normal mouse serum as above. Surface markers used were CD3ε-biotin PPT3 (Abcam), CD4 clone 74-12-4 PerCpCy5.5, CD8α-FITC 76-2-11 (all BD Biosciences), and Near-Infrared Fixable LIVE/DEAD stain (Invitrogen). Biotinylated CD3 was visualized with a streptavidin AF647 (BioLegend). Cells were permeabilized using Cytofix/Cytoperm before intracellular staining with IFN-γ PE P2G10 (BD Biosciences) and cross-reactive anti-human TNF-α-AF650 Mab11 (BioLegend). Samples were fixed in 1% paraformaldehyde before analysis using an LSRFortessa.

#### Statistical analysis.

One-way or two-way ANOVA with Dunnett posttest for multiple comparisons were used to compare immunized groups to the control group, and analysis was performed using GraphPad Prism 6.

## Results

### Viral load and lung pathology in pigs

Groups of six pigs were immunized twice 3 wk apart with an inactivated virus with adjuvant i.m. (Homologous inactivated) or with H3N2 S-FLU by aerosol (S-FLU). The control group was unimmunized (control). Aerosol immunization was carried out using a purpose-built mask and Aerogen Solo nebulizer that allowed efficient vaccine delivery in <5 min to the sedated animals after we had established that the nebulizer did not affect the titer of the S-FLU vaccines and provided a droplet size appropriate for delivery to the lower respiratory tract (Supplemental Fig. 1). Four weeks after the second immunization, the animals were challenged intranasally with swine isolate of pandemic H1N1 A/Sw/Eng/1353/09 (1353/09pdmH1N1) virus and sacrificed 5 dpc.

**FIGURE 1.**
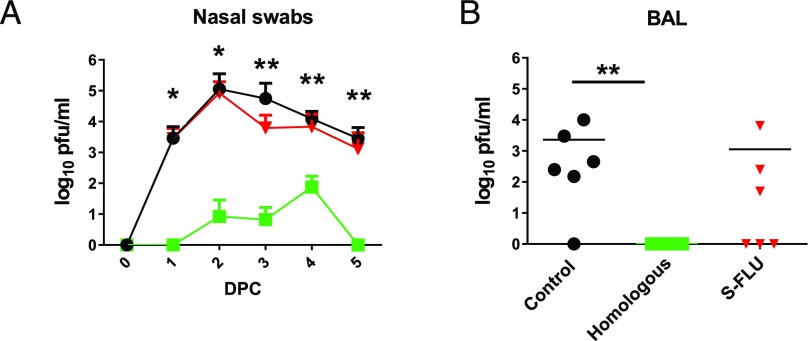
Viral load in nasal swabs. Pigs were immunized twice 21 d apart with either homologous inactivated vaccine by the i.m. route (Homologous) or H3N2 S-FLU by aerosol (S-FLU). Controls were unimmunized pigs. Animals were challenged with 1353/09pdmH1N1 28 or 30 d after the boost. Nasal swabs were taken at 0, 1, 2, 3, 4, and 5 dpc, and pigs were sacrificed at 5 dpc. As the analysis of samples from pigs challenged at days 28 and 30 did not show any significant differences, for simplicity in presentation, the results of the assays carried out on pigs challenged on both days have been amalgamated in this and other figures. Viral titers in the nasal swabs (**A**) and BAL (**B**) were determined by plaque assay. The mean value for shedding for each group is shown over the 5 d (A). Each data point represents an individual within the indicated group, and bars represent the mean (B). Asterisks denote significant differences between the indicated groups and controls. *p < 0.05, **p < 0.01, determined using one-way ANOVA with Dunn test for multiple comparison.

The pigs immunized with the Homologous inactivated vaccine showed the greatest and statistically significant reduction of challenge virus in the nasal swabs at 1, 2, 3, 4, and 5 dpc (Fig. 1A). S-FLU did not reduce viral shedding in nasal swabs significantly at any day postchallenge (Fig. 1A), although two out of the six S-FLU–immunized animals did not shed virus on day 5. No virus was detected in the BAL of the Homologous inactivated vaccine group, and although S-FLU reduced viral load in the BAL, with no virus in three animals, this reduction was not statistically significant (Fig. 1B).

**FIGURE 2.**
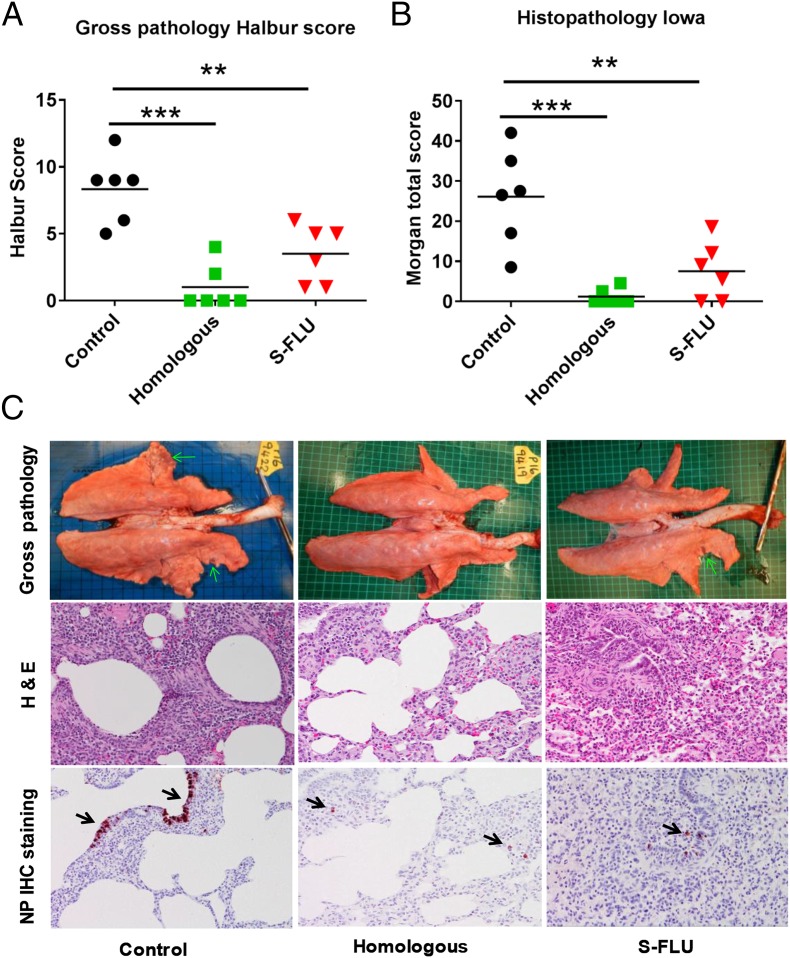
Gross and histopathology. Pigs were immunized twice 21 d apart with either homologous inactivated vaccine i.m. (Homologous) or H3N2 S-FLU by aerosol (S-FLU). Controls were unimmunized animals. Animals were challenged with 1353/09pdmH1N1 on 28 or 30 dpb. Animals were sacrificed at 5 dpc, and lungs were scored for appearance of gross pathology (**A**) and histopathological lesions (**B**). Each data point represents an individual within the indicated group, and bars represent the mean. (**C**) Gross pathology, histopathology (H&E), and immunohistochemical NP staining of representative lungs for each group are shown. Areas of purple-red consolidation (green arrows) are present in lungs from infected groups. Microscopic lesions include alveolar septal inflammation, peribronchiolar inflammatory cell cuffing, and necrotizing/suppurative bronchiolitis with presence of NP Ag in bronchiolar epithelial cells and inflammatory cells (black arrows). Original magnification ×400. Asterisks denote significant differences from the control group. **p < 0.01, ***p < 0.005, determined using one-way ANOVA with Dunn test for multiple comparisons.

Following challenge, the unimmunized animals developed typical gross lesions of influenza virus infection (17). Histopathology showed lesions consisting of severe multifocal interstitial pneumonia, attenuation of the bronchial and bronchiolar epithelium, presence of inflammatory infiltrates within the interalveolar septa and the alveolar lumen, and edema. Immunohistochemical detection of influenza virus NP showed many positive cells within the endothelium of bronchi and bronchioles (Fig. 2).

Animals immunized with the Homologous inactivated vaccine showed very few gross pathological lesions. Histologically, only a few lung sections showed mild interstitial pneumonia and necrosis of the bronchial and bronchiolar epithelium. Virus NP immunostaining was restricted to very few inflammatory cells within the interalveolar septa. The S-FLU–immunized animals showed small areas of gross pathology. Histopathology showed mild to moderate interstitial pneumonia, edema, and epithelial necrosis within the bronchi and bronchioles. Few bronchiolar epithelial and inflammatory interstitial cells exhibited NP immunostaining (Fig. 2).

These results indicated that immunization of pigs with a group 2 H3N2 S-FLU significantly reduced gross and histopathology but did not significantly reduce the viral load in nasal swabs and BAL after heterologous challenge with group 1 H1N1pdm09 virus.

### Ab and IFN-γ ELISPOT responses in pigs

**FIGURE 3.**
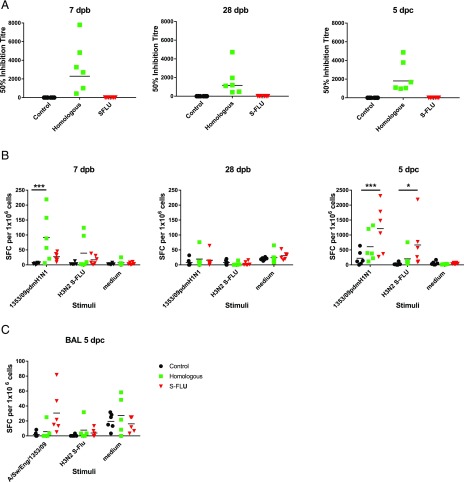
Ab and IFN-γ ELISPOT responses in pigs. Pigs were immunized twice 21 d apart with either homologous inactivated vaccine i.m. (Homologous) or S-FLU by aerosol (S-FLU). Animals were challenged with 1353/09pdmH1N1 on 28 or 30 d after the boost. (**A**) Individual 50% inhibition titers in the serum at 7 dpb, 28 dpb, just before the challenge, and 5 dpc. Numbers of IFN-γ SFC in PBMC (**B**) and BAL (**C**) were determined by ELISPOT following stimulation with the challenge virus 1353/09pdmH1N1 or H3N2 S-FLU in vitro. Results for 1353/09pdmH1N1 and H3N2 S-FLU stimulation were expressed as number of IFN-γ–producing SFC per 10^6^ cells after subtraction of the average number of IFN-γ^+^ cells in medium control wells. Cells cultured in medium alone are also shown to indicate the background obtained. Each data point represents an individual within the indicated group. Asterisks denote significant differences from the control group. *p < 0.05, ***p < 0.005, determined using two-way ANOVA with Dunnett test for multiple comparisons.

We determined the Ab response in pigs using microneutralization assay. Sera from the Homologous inactivated vaccine group had neutralizing Ab, with mean inhibitory titers of 1:2291 at 7 dpb, 1:1166 at 28 dpb, and at 1:1801 at 5 dpc. This indicates that the Homologous inactivated vaccine was successfully delivered and induced anti-1353/09pdmH1N1 neutralizing Abs as expected. Also as expected, no neutralizing Ab to the H1N1 virus was detected in the S-FLU or in the unimmunized controls (Fig. 3A).

We determined influenza-specific T cell responses in PBMC in pigs by IFN-γ ELISPOT at 7 and 28 dpb, just before the challenge, and at the time of sacrifice 5 dpc. PBMC were stimulated with either the challenge virus 1353/09pdmH1N1 or with H3N2 S-FLU. Both homologous inactivated vaccine and S-FLU–immunized animals showed a virus-specific response to the challenge virus at 7 dpb, which was higher in the homologous inactivated group (mean 91 for the homologous inactivated vaccine and 27 for S-FLU spot-forming cells [SFC] per 10^6^ cells). The response to stimulation with H3N2 S-FLU was minimal (mean 39 SFC in homologous inactivated and 17 SFC in S-FLU group). At 28 dpb, just before the challenge, the response was undetectable in any of the groups. At 5 dpc, the S-FLU–immunized animals showed the strongest response to both 1353/09pdmH1N1 and H3N2 S-FLU stimulation (mean 665 and 1211 SFC per 10^6^ cells for homologous inactivated vaccine and S-FLU groups, respectively) (Fig. 3B). The reduced response in the homologous inactivated vaccine group was most likely due to the lack of Ag stimulation because of the greatly reduced influenza A viral load in these animals.

The response in the BAL showed a similar trend. However, the detectable response was apparently much weaker (∼30 SFC per 10^6^ cells for S-FLU) because of the low percentage of T cells in BAL. There was also a high medium control background most likely because of the presence of many activated cells in the BAL (Fig. 3C). These data show that, as expected, the Homologous inactivated vaccine induced a strong Ab response, whereas in contrast, S-FLU did not induce detectable neutralizing Ab, but these animals had the highest number of IFN-γ–producing cells following stimulation with either 1353/09pdmH1N1 or H3N2 S-FLU postchallenge.

### Analysis of cytokine-producing cells

**FIGURE 4.**
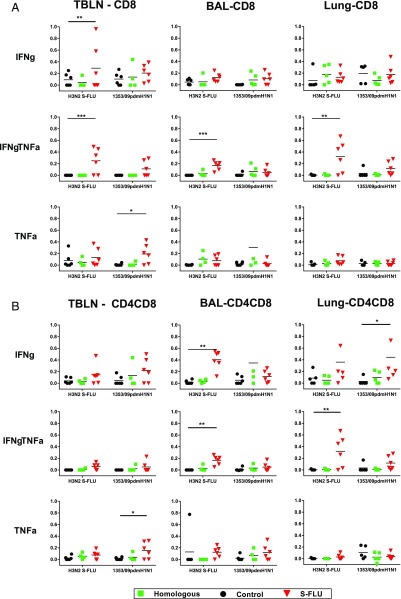
Cytokine-producing cells in pig TBLN, BAL, and lung tissues. Flow cytometry was used to quantitate the frequency of IFN-γ□, IFN-γ TNF-α–, and TNF-α–positive cells within CD8hi (**A**) and CD4+CD8+ (**B**) cells at 5 dpc. Cells were stimulated with either challenge virus 1353/09pdmH1N1 or H3N2 S-FLU. Each data point represents an individual within the indicated group. Asterisks denote significant differences from the control group. *p < 0.05, **p < 0.01, ***p < 0.005, determined using two-way ANOVA with Dunnett test for multiple comparisons.

To dissect which cells produce cytokines, we performed intracellular staining for IFN-γ and TNF-α combined with surface staining for CD4, CD8, and CD4CD8 cell subsets. The latter are the activated memory CD4 cells in pigs (18). To analyze local immune responses, TBLN, lung, and BAL cells were stimulated with either the challenge virus 1353/09pdmH1N1 or with H3N2 S-FLU. S-FLU immunization induced the highest proportion of double IFN-γ TNF-α cytokine-producing CD8 cells in lymph node, BAL, and lung, followed by single IFN-γ or single TNF-α producers in the TBLN (Fig. 4A). Similarly, the S-FLU–immunized animals had the highest proportion of CD4CD8 cells producing single IFN-γ and double IFN-γ TNF-α cytokines in the BAL and lung (Fig 4B). A statistically significant proportion of single TNF-α producers was observed in TBLN CD8 and CD4CD8 cells. The CD4 response was negligible in all tissues and is not shown. In contrast to the local tissues, systemic responses analyzed in PBMC and spleen showed lower proportions of CD8 or CD4CD8 Ag-specific cells (data not shown).

In summary, the S-FLU immunization induced a strong local lung response to IAV and S-FLU. The high frequency of these single and double IFN-γ and TNF-α producers may account for the protective efficacy of local immunization.

### Tissue-resident memory cells

**FIGURE 5.**
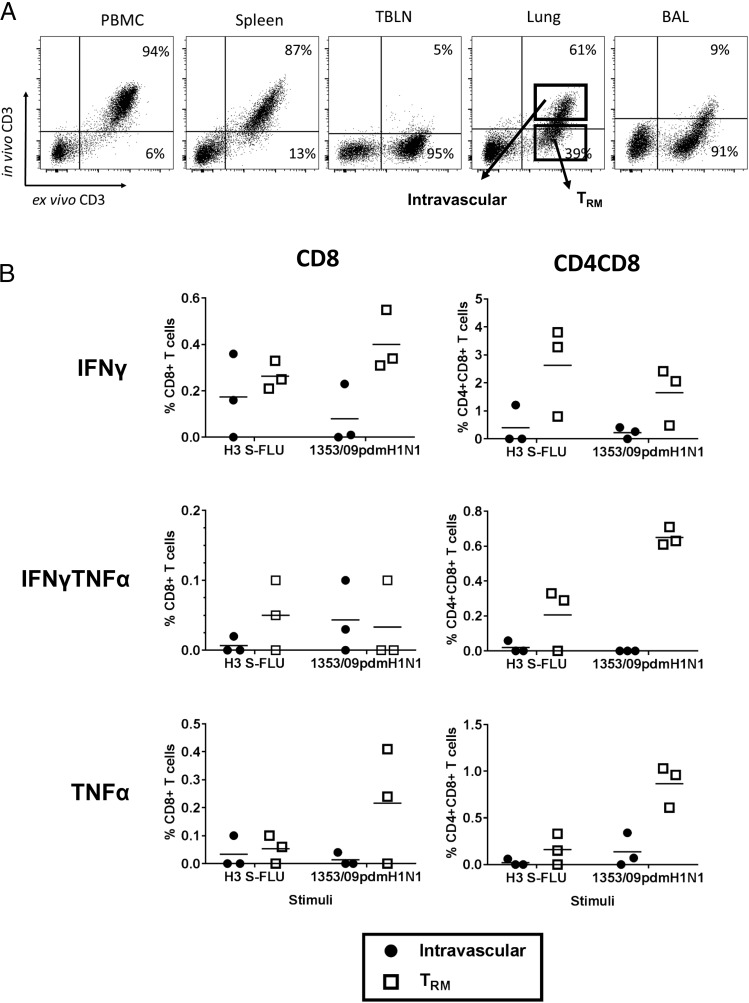
Porcine lung T_RM_. (**A**) Before sacrifice, three pigs from each group were infused i.v. with CD3 Ab and sacrificed 10 min later. Lymphocytes were isolated and stained ex vivo with anti-mouse Ig-FITC, and the same CD3 Ab labeled with PeCy5. As the infused CD3 does not saturate all CD3 sites, blood, spleen, and some lung tissue T cells are double positive (intravascular cells). BAL and some of lung cells are unstained by intravascular Ab (T_RM_). (**B**) Lower panels show IFN-γ and TNF-α production by intravascular and T_RM_ CD8 and CD4CD8 cells in H3N2 S-FLU–immunized animals after in vitro stimulation with either challenge virus 1353/09pdmH1N1 or H3N2 S-FLU.

Because we have shown that aerosol immunization with H3N2 S-FLU induced a strong local immune response, we wished to establish whether the responding cells were part of the T_RM_ population. To distinguish T_RM_ in the lungs of pigs from circulating cells present in the vasculature of the tissue, we administered CD3 mAb i.v. 10 min before sacrificing the animal. After sacrifice, the lymphocytes were isolated and stained ex vivo with anti-mouse IgG-FITC and with the same clone of CD3 directly labeled with PeCy5. As the infused CD3 does not saturate all CD3 sites, blood, spleen, and some lung tissue T cells are double positive (intravascular cells) (Fig. 5A). In contrast, most BAL and some lung tissue cells are only CD3PeCy5 positive, indicating that they are inaccessible to the Ab in the blood (T_RM_). Lymph nodes were inaccessible to the infused CD3 Ab, as has been shown to be the case by others (19). There was no difference in the proportions of T_RM_ and intravascular cells in the immunized and control pigs (data not shown). A similar pattern has been observed in more than 20 animals from other studies.

As BAL is 90% stained only by the ex vivo CD3PeCy5, whereas the blood and spleen are double labeled, this indicates that most BAL cells are part of the blood-inaccessible pool of T_RM_. Because BAL gives a strong Ag-specific response, we can conclude that S-FLU is inducing lung T_RM_. Our staining in lung indicates that a proportion of lung T cells (39%) are inaccessible to i.v. CD3 Ab and are therefore also part of the T_RM_ population. The T_RM_ in the three S-FLU–immunized animals treated with i.v. Ab had a higher proportion of Ag-specific cells producing IFN-γ and TNF-α than the lung intravascular population (Fig. 5B) following stimulation with either the challenge virus 1353/09pdmH1N1 or with H3N2 S-FLU. The comparison between intravascular and T_RM_ in the Homologous inactivated and control groups is unreliable because there were few responding cells and therefore very few events in the gated populations (data not shown). These results demonstrate that aerosol immunization with H3N2 S-FLU induces a large lung T_RM_ population.

### Evaluation of the H3N2 S-FLU vaccine in ferrets

**FIGURE 6.**
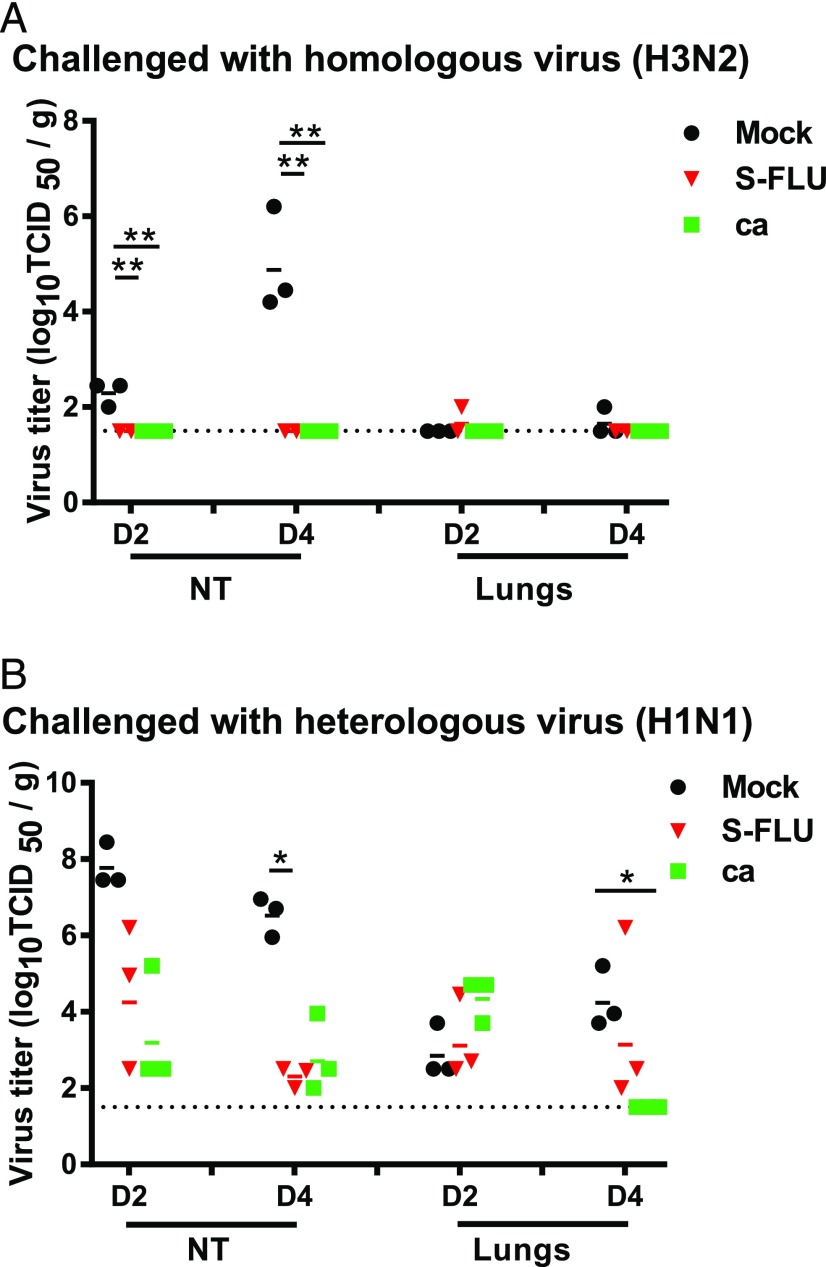
Virus replication in respiratory tissues of ferrets immunized and challenged with homologous or heterologous virus. Ferrets were lightly anesthetized with isoflurane and immunized intranasally with two doses of 10^7^ TCID_50_ in 0.5 ml of A/Switzerland/9715293/2013 S-FLU, 10^7^ TCID_50_ in 0.5 ml of A/Switzerland/9715293/2013 ca, or 0.5 ml of L-15 21 d apart. The ferrets were challenged with 10^6^ TCID_50_ in 1 ml of influenza A/Switzerland/9715293/2013 (H3N2) (**A**) or A/California/07/2009 (H1N1pdm09) (**B**) virus. Ferrets from each group were sacrificed on days 2 (D2) and 4 (D4) postinfection, and viral load in their lungs and NT was determined and expressed as log_10_ TCID_50_ per g. Dotted lines indicate the limit of detection for each assay. Horizontal bars represent mean titers, and symbols represent titers from individual ferrets. *p < 0.05, **p < 0.002.

We next determined the protective efficacy of the same batch of H3 S-FLU in ferrets, using as a positive control live attenuated virus H3N2 ca, as we have previously used similar ca viruses for this purpose in ferrets (5). We also immunized intranasally rather than by aerosol as, in anesthetized small animals, intranasal administration has been shown to reach the lungs (20, 21). Groups of 12 ferrets were immunized intranasally twice with H3N2 S-FLU or H3N2 ca, and 12 ferrets were mock-vaccinated. Three weeks later, half the animals in each group (*n* = 6 per group) were challenged with intranasally delivered homologous wild-type (wt) influenza A/Switzerland/9715293/2013 (H3N2) and the other half with a heterologous influenza A/California/07/2009 (H1N1pdm09) virus. On days 2 and 4 postchallenge, three ferrets in each group were sacrificed, and virus titers in lungs and NT were assessed. The homologous wt H3N2 virus did not replicate in the lower respiratory tract of mock-immunized (or vaccinated) ferrets (Fig. 6A), so the efficacy of the vaccines in protecting against pulmonary virus replication could not be assessed. However, wt H3N2 virus replicated to a moderately high titer (mean 10^4.95^TCID_50_/g) in the NT at 4 dpc, and both vaccines prevented replication of the challenge virus in the NT (*p* < 0.05).

The H1N1pdm09 virus that was used for heterosubtypic challenge replicated to a high titer in the NT (mean 10^7.8^ and 10^6.5^ TCID_50_/g at 2 and 4 dpc, respectively) and to a modest-to-moderate titer (mean 10^2.9^ and 10^4.3^ TCID_50_/g at 2 and 4 dpc) in the lungs (Fig. 6B). Both the S-FLU and ca vaccine viruses provided modest reduction (10^4.6^ and 10^3.4^ TCID_50_/g, respectively) in H1N1pdm09 titers in the NT at 2 dpc and further reduction (10^2.3^ and 10^2.8^, respectively) at 4 dpc, although only the S-FLU group on day 4 was significantly different from the mock-immunized group (*p* < 0.05). A statistically significant reduction in lung virus titers was observed on day 4 postchallenge but only in animals that had received the H3N2 ca virus vaccine (*p* = 0.002).

### Effect of H3N2 S-FLU vaccine on transmission in ferrets

We next determined whether immunization with H3N2 S-FLU would prevent transmission of the heterologous H1N1pdm09 challenge virus. Four ferrets each were vaccinated intranasally with two doses of H3N2 S-FLU, and four ferrets were mock vaccinated; the ferrets were challenged intranasally with influenza A/California/07/2009 (H1N1pdm09) 21 d after the second dose of vaccine and were placed in transmission cages. The following day, an unvaccinated naive ferret was introduced adjacent to each infected ferret. The experimentally infected and respiratory contact ferrets were followed with nasal washes and serology to determine whether the H1N1pdm09 virus transmits by the respiratory route from experimentally infected to airborne contact ferrets. One ferret each in the mock-immunized and H3N2 S-FLU–vaccinated groups reached their humane endpoints 9 dpc from an intercurrent infection in the animal house. Unfortunately, we were not able to identify the etiology of the intercurrent infection that caused weight loss in ferrets. None of the ferrets were found dead. They were euthanized in accordance with our approved animal study protocol because of weight loss. The virus inoculum was not contaminated with bacteria, and the virus dose was confirmed to be correct.

**FIGURE 7.**
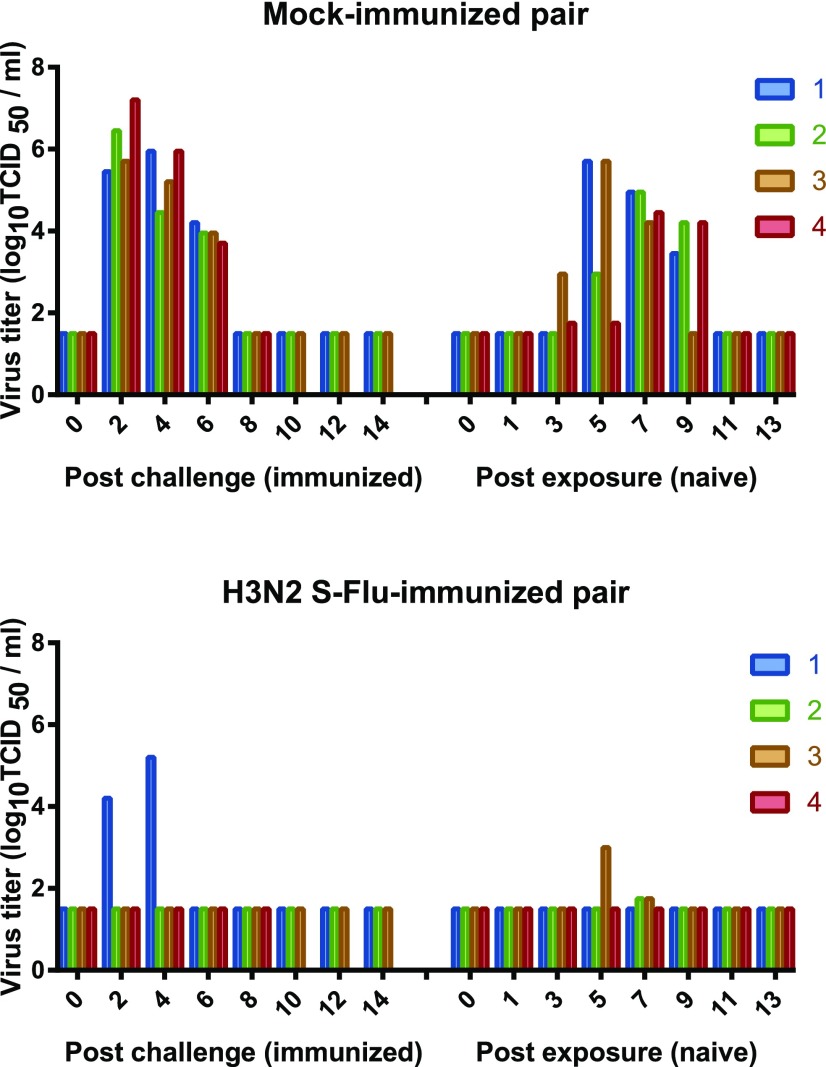
Ferrets immunized with H3N2 vaccine were protected against challenge infection with H1N1pdm09 virus, and the transmission to naive animals was restricted. Four ferrets were vaccinated intranasally with two doses of H3N2 S-FLU, and four ferrets were mock-vaccinated. Twenty-one days after the second immunization, the ferrets were challenged intranasally with influenza A/California/07/2009 (H1N1pdm09), and the ferrets were placed in transmission cages. The following day, an unvaccinated naive ferret was introduced adjacent to each infected ferret. Nasal washes were collected every other day for 14 d, and virus titers in the experimentally infected and airborne contact ferrets are presented. Each bar represents a single ferret. One ferret each in the mock-immunized and H3N2 S-FLU–vaccinated group reached their humane endpoints 9 dpc from an intercurrent infection in the animal house (etiology not identified).

All four of the mock-immunized ferrets were infected with H1N1pdm09 and transmitted to their airborne contacts (Fig. 7). Infection with and transmission of the H1N1pdm09 virus was greatly reduced in frequency and viral load in the animals that were immunized with H3N2 S-FLU vaccine and their airborne contacts. Challenge virus was detected in nasal washes of one of three H3N2 S-FLU–vaccinated ferrets, but all three seroconverted. Virus was detected in two of three of the corresponding airborne contact animals at a low titer for a limited period, but neither of the contact animals seroconverted. Thus, the H3N2 S-FLU vaccine severely restricted shedding of the H1N1pdm09 challenge virus, although infection did occur. Although airborne transmission occurred, the intensity of the transmitted infection to airborne contact ferrets was markedly reduced with low titer, short duration viral shedding, and no seroconversion in airborne contact ferrets.

## Discussion

There is strong epidemiological evidence for an association between a cross-reactive T cell response and heterologous protection between group 1 and 2 IAV in humans (22, 23). In the first of these studies, the association with the T cell response was predominantly with reduced fever and symptoms (22), whereas in the second, the T cell response to NP was associated with reduced viral shedding in symptomatic volunteers (23). In these studies, the correlation was with the T cell response in peripheral blood that had likely been induced by prior natural infection. This evidence, combined with a long history of animal studies demonstrating the protective effect of T cells induced by live influenza virus infection (24), provides the rationale for developing a safe and BPIV that induces a strong T cell response in the human lung. In this study, we compared the outcome of heterosubtypic influenza virus challenge after S-FLU vaccination in both pigs and ferrets. H3N2 S-FLU immunization of pigs had a minimal effect on H1N1pdm09 replication after challenge but a significant effect on pathology. By contrast, in the ferret, the same vaccine preparation induced sterile immunity to the matched H3N2 challenge and reduced replication and aerosol transmission to naive recipients following H1N1pdm09 challenge. Our results also show that aerosol delivery of H3N2 S-FLU vaccine is safe and induced strong local lung immune responses and T_RM_ in BAL and lung tissues of pigs.

In earlier experiments in ferrets and mice, in which there was a complete mismatch between the immunizing H1N1 or H5N1 (group 1) S-FLU and challenge H3N2 and H7N9 (group 2), we observed a significant reduction in replication of the infectious challenge virus (3, 5). In the current study, we used another mismatched immunization and challenge combination and confirmed a significant effect on replication of the challenge virus in ferrets but not pigs. A possible reason for the difference between pigs and ferrets might be that the H3N2 S-FLU used in this study is coated with the clade 3C.3a H3 HA. This H3 is exquisitely specific for α2–6 sialic acid but has low affinity (25) and, although the pig respiratory tract expresses both α2–3 and 2–6 (26), it is possible that the binding of H3 to the pig respiratory tract is poor. Experiments with S-FLU coated in different HAs may resolve this. Another possibility is that, although H3N2 S-FLU induced a strong local response against the immunizing and challenge viruses, this was insufficient to prevent replication of the challenge virus. We speculate that a higher dose of vaccine might be required, as earlier work in pigs immunized with attenuated influenza showed a reduction in challenge virus replication despite mismatching (27). Further work to examine whether a higher dose of vaccine is required to fully protect the lungs of large animals needs to be performed.

In other experiments in small animals, the effect of fully heterosubtypic immunization was similar to what we observe in pigs. For example, a single-replication cycle H1N1 (group 1) BPIV based on the partial deletion of the M2 gene (28) induced sterilizing immunity against matched challenge and protected mice against death from heterosubtypic H3N2 (group 2) challenge but did not prevent viral replication in NT and lung. The protective effect was associated with the induction of cross-reactive T cells but not Ab and a reduced inflammatory neutrophil infiltrate in the lung. In ferrets with partial matching of a single-cycle live attenuated virus, in which vaccine (H1N1, group 1) and challenge (H5N1, group 1) were selected from the same genetic group, protection was associated with the induction of cross-reactive Ab to the conserved group 1 HA stem and the N1 NA in addition to cross-reactive T cells, and challenge viral replication was reduced (29). These results suggest that protective immune responses to live attenuated or single-cycle viruses may be cumulative, and partial matching between vaccine and challenge within the same genetic group can add incremental protective value through the induction of cross-reactive Abs, as strongly suggested by the epidemiology of human infections (30). Unfortunately, the group of origin of future pandemic or even seasonal viruses cannot be predicted.

It is increasingly evident that local immune responses and particularly lung T_RM_ play a major role in protection against influenza viruses in mice (9, 10, 31). Pulmonary T_RM_ in the BAL and lung tissues have greater protective capacity than circulating memory CD8 T cells (9, 32, 33). BAL T_RM_ are associated with reduced symptoms and viral load in respiratory syncytial virus infection in humans (34). To our knowledge, in this study we show for the first time that we can distinguish T_RM_ in pigs, as has been shown in mice following i.v. administration of CD3 Ab. We demonstrate that more than 90% of BAL cells are inaccessible to intravascular Ab as well as a proportion of the lung tissue cells. Aerosol immunization with H3N2 S-FLU induces a strong immune response of these cells, which may not reduce viral replication, but may be able to induce a beneficial reduction in local inflammation through the release of immuno-modulating cytokines (35–37).

In summary, our data show that the same vaccine has different protective efficacy in pigs and ferrets. In the absence of Ab, lung T cell immunity can consistently reduce disease severity but does not always abolish viral replication. We suggest that candidate BPIV should be tested in more than one species. The pig maybe a relevant large animal model because it is a natural host for influenza viruses and has very similar respiratory anatomy to humans.

## Supplementary Material

Data Supplement
