# Supplementary material for: Comparison of Heterosubtypic Protection in Ferrets and Pigs Induced by a Single-Cycle Influenza Vaccine
Source: J Immunol. 2018 Apr 27;200(12):4068–77. doi: 10.4049/jimmunol.1800142 (PMC5985365; doi:10.4049/jimmunol.1800142)
Supplement: Data Supplement [file JI_1800142.zip › JI_1800142_Supplemental_Figure_1.pdf]

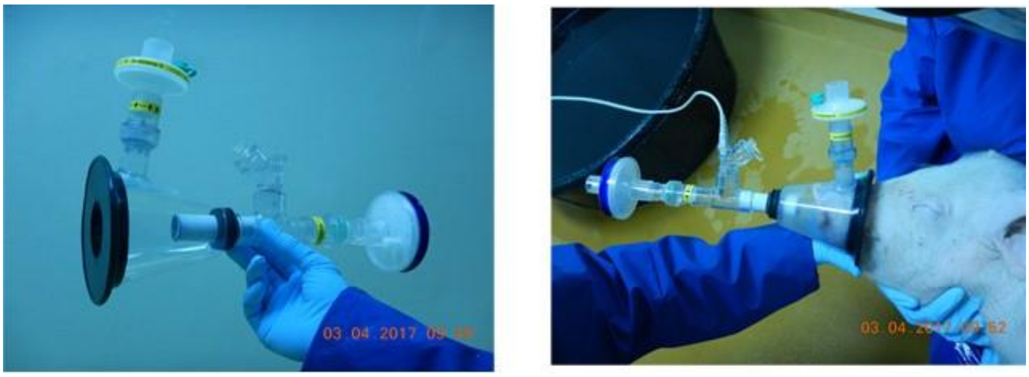

Supplemental Figure. Aerosol delivery of H3N2 S-FLU in pigs. Custom built mask for aerosol delivery in pigs.
